# Breast cancer incidence and mortality in women in China: temporal trends and projections to 2030

**DOI:** 10.20892/j.issn.2095-3941.2020.0523

**Published:** 2021-08-15

**Authors:** Shaoyuan Lei, Rongshou Zheng, Siwei Zhang, Ru Chen, Shaoming Wang, Kexin Sun, Hongmei Zeng, Wenqiang Wei, Jie He

**Affiliations:** 1 Office for Cancer Registry, National Cancer Center/National Clinical Research Center for Cancer/Cancer Hospital, Chinese Academy of Medical Science and Peking Union Medical College, Beijing 100021, China; 2 Department of Thoracic Surgery, National Cancer Center/National Clinical Research Center for Cancer/Cancer Hospital, Chinese Academy of Medical Science and Peking Union Medical College, Beijing 100021, China

**Keywords:** Breast cancer, temporal trends, prediction, cancer registry, China

## Abstract

**Objective:**

Breast cancer was the most common cancer and the fifth cause of cancer deaths among women in China in 2015. The evaluation of the long-term incidence and mortality trends and the prediction of the future burden of breast cancer could provide valuable information for developing prevention and control strategies.

**Methods:**

The burden of breast cancer in China in 2015 was estimated by using qualified data from 368 cancer registries from the National Central Cancer Registry. Incident cases and deaths in 22 cancer registries were used to assess the time trends from 2000 to 2015. A Bayesian age-period-cohort model was used to project the burden of breast cancer to 2030.

**Results:**

Approximately 303,600 new cases of breast cancer (205,100 from urban areas and 98,500 from rural areas) and 70,400 breast cancer deaths (45,100 from urban areas and 24,500 from rural areas) occurred in China in 2015. Urban regions of China had the highest incidence and mortality rates. The most common histological subtype of breast cancer was invasive ductal carcinoma, followed by invasive lobular carcinoma. The age-standardized incidence and mortality rates increased by 3.3% and 1.0% per year during 2000–2015, and were projected to increase by more than 11% until 2030. Changes in risk and demographic factors between 2015 and 2030 in cases are predicted to increase by approximately 13.3% and 22.9%, whereas deaths are predicted to increase by 13.1% and 40.9%, respectively.

**Conclusions:**

The incidence and mortality of breast cancer continue to increase in China. There are no signs that this trend will stop by 2030, particularly in rural areas. Effective breast cancer prevention strategies are therefore urgently needed in China.

## Introduction

Breast cancer is the most commonly diagnosed cancer and the leading cause of cancer deaths among women worldwide^1^. According to GLOBOCAN 2018^1^, there were 2.1 million newly diagnosed cases and 627,000 deaths of breast cancer worldwide in 2018. Wide variation was observed across geographic regions, and the incidence in developed regions was much higher than that in less developed regions^1^. China, the largest developing country, has a relatively low incidence of breast cancer worldwide, but this incidence has increased more than twice as rapidly as the global rate since 1990s, particularly in urban areas^2^. Breast cancer is now the most common cancer and the fifth cause of cancer deaths among Chinese women, according to data from 2015^3^. With rapid economic development, aging and growth of the population, as well as an increasing prevalence of the main risk factors^4–9^ the burden of breast cancer may continue to grow in the future. Assessment of breast cancer trends and projection of the future burden would provide valuable information for cancer control strategies. However, most related studies have focused on only changes in mortality or only certain regions in China^10,11^, which cannot fully represent the national epidemic of breast cancer. Established in 2002, the National Central Cancer Registry (NCCR) of China is an information system for cancer data collection, management, and analysis. It provides up-to-date qualified cancer data, which include incidence, mortality, and survival, and are the most representative data currently available in China. On the basis of the NCCR data, we provide the estimated numbers of new breast cancer cases and deaths; the distribution of histological subtypes in 2015 in China at the national level; the temporal trends in past decades; and predictions to 2030. This information may not only help policy makers develop better intervention strategies and reallocate medical resources, but also provide a reference for other rapidly developing countries.

## Materials and methods

### Data source and quality control

The NCCR is responsible for cancer data collection from local population-based cancer registries, and their evaluation, and publication. Cancer related information is reported to registries from local hospitals and community health centers, including Basic Medical Insurance for Urban Residents and the New-Rural Cooperative Medical System. The Vital Statistical Database is linked to the cancer incidence database to identify cases with a death certificate only (DCO) and follow-up. Quality control is conducted according to the criteria of the Chinese Guidelines for Cancer Registration and Cancer Incidence in Five Continents (CI5). Detailed information has been published in a previous study^12^. Briefly, the completeness, comparability, and validity of the data were evaluated according to indicators, such as the mortality to incidence (M/I) ratio, the percentage of cases morphologically verified (MV%), the percentage of death certificate-only cases (DCO%), and the stability of cancer trends over the years, as provided in the appendix (**Supplementary Table S1**). All cancer cases and deaths were classified according to the International Classification of Diseases for Oncology, 3rd edition (ICD-O-3) and the International Statistical Classification of Diseases and Related Health Problems 10th Revision (ICD-10).

In this study, ICD-10 data code C50 was extracted from the overall cancer database to estimate the breast cancer incidence and mortality rates. According to the ICD-O-3 morphological code, breast cancer was classified as invasive ductal carcinoma (8,500/3), lobular invasive carcinoma (8,520/3, 8,521/3, 8,522/3), Paget’s disease (8,540/3, 8,541/3, 8,542/3, 8,543/3), medullary carcinoma (8,510/3, 8,512/3, 8,513/3), unspecified type (8,000/3, 8,010/3 or with missing data), and other rare types with small numbers of cases.

### Statistical analysis

A total of 368 cancer registries with qualified cancer statistic data were used to describe the breast cancer incidence and mortality in 2015 (**Supplementary Figure S1**). Crude incidence and mortality rates were calculated for different areas (urban/rural) and age groups (0, 1–4, 5–80 in increments of 5 years, and 85+). The numbers of new cases and deaths were estimated from the age-specific cancer incidence or mortality rates from 368 cancer registries, multiplied by the corresponding populations. The incidence and mortality rates were standardized by using Segi’s world standard population^13^.

We determined the incident cases and deaths in 22 cancer registries covering approximately 3.34% of China’s national population, and used continuous qualified data to assess the time trends from 2000 to 2015. Data from 2008 to 2015 were used to analyze breast cancer histological subtype trends. Annual percentage changes were estimated with Joinpoint regression (version 4.3.1.0, https://surveillance.cancer.gov/joinpoint/). To reduce the possibility of reporting spurious changes in trends over the period, we restricted models to a maximum of 2 joinpoints. Trends are expressed as annual percentage change (APC), and the Z test was used to assess whether the changes were statistically different from zero.

The burden of breast cancer in 2030 was projected for each age group and area by using the Bayesian age-period-cohort method. The traditional age-period-cohort model can be formulated as:

$$y_{ij}\sim B(n_{ij},p_{ij})$$

$$n_{ij}=\log\left(\frac{p_{ij}}{1-p_{ij}}\right)=\mu +\theta_{i}+\Phi_{j}+\psi_{k}$$

The logit of the incidence probability consists of an intercept μ, age effect θ*_i_*, period effect Φ*_j_*, and cohort effect ψ*_k_*. The Bayesian hierarchical approach uses Gaussian random walk (RW) priors of different orders for the APC parameters of θ, ϕ, and ψ^14,15^. It combines prior knowledge with observed data to derive a posterior distribution. The RW1 prior was used to predict the breast cancer incidence rate and the RW2 prior was used to predict the mortality rate. This model was conducted in the software package Bayesian Age-Period-Cohort Modeling and Prediction package (BAMP v.1.3.0, Institute of Biomedical Engineering, Imperial College, London, UK)^16^. Markov chain Monte Carlo simulations were run for 1,010,000 iterations, with the initial 10,000 iterations used as burn-in to minimize the effects of initial values. The projected rates p_25_, p_50_ and p_95_ were obtained through 1,000,000 iterations of model simulations. The posterior and predictive deviance of the model were used as a measure of goodness of fit^17^.

To forecast the number of new cases or deaths, we multiplied the projected rate p_50_ by China’s projected population from World Population Prospects 2019 (https://population.un.org/). We also divided the predicted breast cancer cases and deaths into contributions from changes in risk and changes in population, including the population size and age structure, according to methods described by Moller et al.^18^

## Results

### Breast cancer incidence and mortality rates in 2015

We estimated approximately 303,600 new breast cancer cases and approximately 70,400 deaths in China in 2015. The crude and age standardized rate of breast cancer incidence were 45.29/100,000 and 29.56/100,000, ranking first in the incidence of cancers in women in China. The crude and age standardized rate of breast cancer mortality were 10.50/100,000 and 6.48/100,000, ranking fifth in the mortality of cancers in women in China. For different regions, both the incidence and mortality rates were higher in urban areas than in rural areas (**Table 1**). Age-specific incidence and mortality are shown in **Supplementary Figure S2**.

**Table 1:** The incidence and mortality of breast cancer in women in China, 2015

Areas	Incidence					Mortality				
	Cases	Crude rate	Proportion	ASIR	Rank*	Deaths	Crude rate	Proportion	ASMR	Rank*
	(*n* × 10^4^)	(1/10^5^)	(%)	(1/10^5^)		(*n* × 10^4^)	(1/10^5^)	(%)	(1/10^5^)	
All areas	30.36	45.29	17.08	29.56	1	7.04	10.50	8.2	6.48	5
Urban areas	20.51	54.31	18.76	33.75	1	4.59	12.16	9.26	7.11	3
Rural areas	9.85	33.64	14.38	23.63	2	2.45	8.37	6.76	5.6	6

ASIR, age-standardized incidence rate using Segi’s world standard population; ASMR, age-standardized mortality rate using Segi’s world standard population. *Rank represents the order of breast cancer incidence/mortality in different areas among all cancers.

### Distribution of histological subtypes of breast cancer in 2015

**Figure 1:**
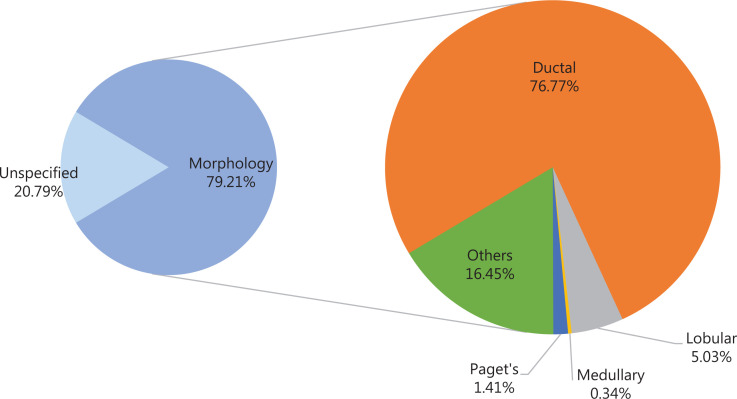
Distribution of histological subtypes of breast cancer in 2015.

**Figure 1** shows the distribution of breast cancer cases in China by histological subtype in 2015. Overall, 79.21% of breast cancer cases had morphological taxonomy, and among those, the most common morphology of breast cancer was invasive ductal carcinoma (76.77%), followed by invasive lobular carcinoma (5.03%). Paget’s disease (1.41%) and medullary carcinoma (0.34%) had a relatively small proportion of breast cancer cases.

### Trends in breast cancer incidence and mortality

#### Incidence trends by age

**Figure 2:**
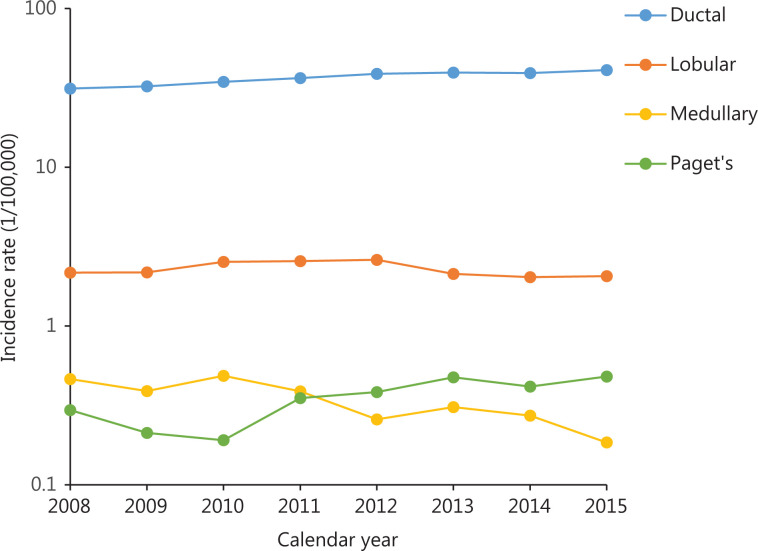
Breast cancer incidence trends by histological subtype during 2008–2015.

During 2000 to 2015, the age-standardized incidence rates of breast cancer increased by approximately 2.6% per year in urban areas and 6.9% per year in rural areas (**Figure 2**, **Table 2**). Analysis of age-specific incidence rates showed the greatest increases in younger age groups (<40 years) in all areas (**Table 2**). The increasing trends were clearer in rural areas and increased by approximately 8.5% in people <40 years of age and 7.0% in people 50–59 years of age in rural areas compared with urban areas (4.3% and 3.0%, respectively). The incidence remained stable among people over 80 years of age in urban areas but continued to show rapid increases in rural areas (**Supplementary Table S2**).

**Table 2:** Trends in age-specific breast cancer incidence and mortality rates in women by area in China, 2000–2015 (%)

Age group	Incidence			Mortality		
	All areas	Urban areas	Rural areas	All areas	Urban areas	Rural areas
ASR	3.3*	2.6*	6.9*	1.0*	0.6*	2.7*
<40	4.8*	4.3*	8.5*	0.9	0.7	1.7
40–49	2.0*	1.5*	6.8*	–1.3*	–1.8*	1.6
50–59	3.8*	3.0*	7.0*	0.7	0.3	2.0*
60–69	3.9*	3.5*	6.0*	1.5*	1.3*	3.5
70–79	3.3*	2.7*	5.0*	2.4*	1.9*	5.8*
80+	0.8	0.1	5.5*	3.8*	3.2*	6.3*

ASR, age-standardized rate using Segi’s world standard population; *, average annual percentage change during 2000–2015 is significantly different from zero (*P* < 0.05).

#### Incidence trends by histological subtype

**Figure 3:**
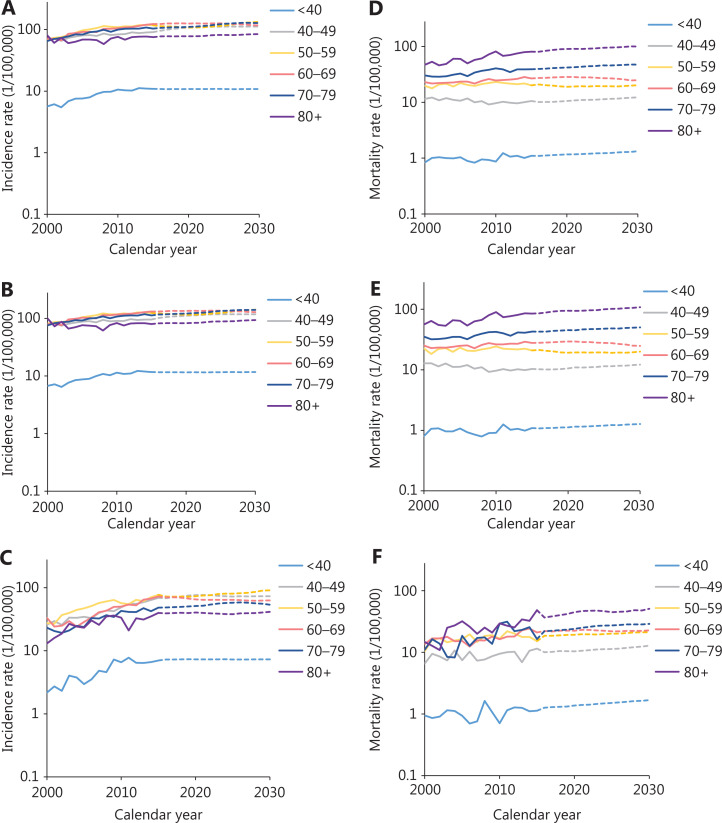
Trends in age-specific incidence and mortality rates during 2000–2015 and predictions from 2016 to 2030 in China. (A) Total incidence; (B) urban incidence; (C) rural incidence; (D) total mortality; (E) urban mortality; (F) rural mortality.

Trends in breast cancer incidence rates by histological subtype are shown in **Figure 3**. During 2008 to 2015, invasive ductal carcinoma and Paget’s disease increased, with an annual increase of 3.9% and 12.1%, respectively, whereas invasive lobular carcinoma showed an annual decrease of 1.1% and 11.2%, respectively (**Supplementary Table S3**).

#### Mortality trends by age

The long-term trends in breast cancer mortality rates were also analyzed by region and age group during 2000–2015 (**Figure 2**, **Table 2**). The age-standardized mortality rate had a slight increase of approximately 0.6% per year in urban areas and 2.6% per year in rural areas. The age-specific mortality rate remained steady in people under 50 years of age in both urban and rural areas. However, the mortality rate trends continued to increase beyond age 70, particularly in rural areas. Further analysis showed an increasing incidence trend in the more recent birth cohort for all age groups, in both urban and rural areas. However, a steady trend in the younger age group was observed in the more recent birth cohort (**Supplementary Figure S3**). More details about the Joinpoint analysis are provided in **Supplementary Table S4**.

#### Projection of numbers of new cases and deaths

**Figure 4:**
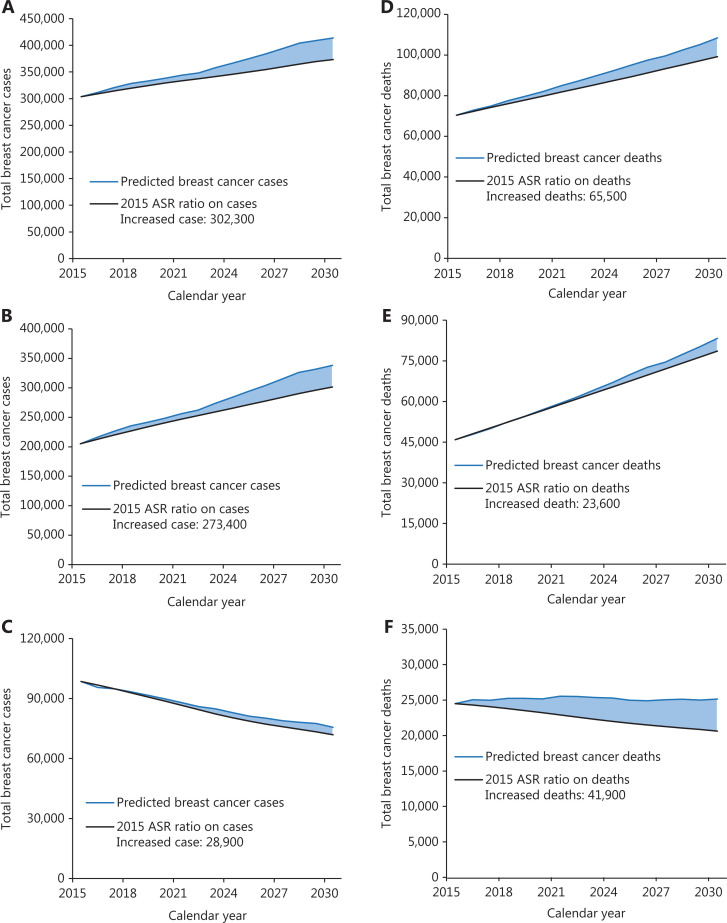
The total increased breast cancer cases and deaths between 2015 and 2030 in China. (A) Total cases; (B) urban cases; (C) rural cases; (D) total deaths; (E) urban deaths; (F) rural deaths; the blue line represents predicted cancer cases or deaths from 2015 to 2030; the black line represents estimated numbers of total breast cancer cases or deaths by application of the 2015 age-specific incidence or mortality rate.

The projections of future trends in breast cancer incidence, mortality, and burden through 2030 by region in China are presented in **Table 3**. The incidence and mortality rates for calendar years 2000–2015 and predicted trends from 2016 to 2030 are shown in **Figure 3**. According to this graph, the forecast incidence and mortality rates will continue to increase in most age groups. The age-standardized incidence and mortality rates in 2030 are expected to increase to 35.95/100,000 and 11.94/100,000. The number of new incident cases of breast cancer increased by approximately 36.27% with the changing age-specific incidence rates (or could be explained by changes due to risk) contributing 13.33%, changing age structures contributing 10.98%, and population growth contributing 11.96% to the overall increase. The number of breast cancer deaths increased by approximately 54.01%, with changing age-specific mortality rates contributing 13.09%, changing age structures contributing 24.10%, and population growth contributing 16.82% to the overall increase (**Table 3**). The estimated number of predicted cancer cases and deaths from 2015 to 2030 due to the rise in rates since 2015 is shown in **Figure 4**. We predicted increases of approximately 302,300 breast cancer cases due to the risk factors (273,400 in urban areas and 28,900 in rural areas) and 65,500 breast cancer deaths (23,600 in urban areas and 41,900 in rural areas) from 2015 to 2030 in China.

**Table 3:** Predicted number of new breast cancer cases and deaths in China and changes between 2015 and 2030, stratified into changes due to risk and demographics by region

Areas	Number of new cases or deaths				Change between 2015 and 2030 (%)				
	2015	2020	2025	2030	Total change	Change due to risk	Change due to demographics	Change due to age structure	Change due to population size
Incidence									
All areas	303,600	338,200	375,100	413,800	36.27	13.33	22.94	10.98	11.96
Urban areas	205,100	248,400	293,900	338,100	64.87	17.92	46.96	11.79	35.17
Rural areas	98,500	89,800	81,200	75,600	–23.26	3.79	–27.05	9.31	–36.36
Mortality									
All areas	70,400	82,100	95,000	108,400	54.01	13.09	40.92	24.10	16.82
Urban areas	45,900	57,000	70,000	83,300	81.45	10.24	71.21	27.80	43.41
Rural areas	24,500	25,200	25,300	25,100	2.60	18.42	–15.82	17.17	–32.99

## Discussion

This study performed an updated systematic analysis of the nationwide disease burden of breast cancer in women in China in 2015, and provides forecasts to 2030. Breast cancer had the highest incidence among cancers in women in China. The age-standardized incidence rate was higher than some of the less developed countries, such as Niger and Tanzania, but much lower than those in developed countries^1^. The highest incidence of breast cancer in China occurred in the socioeconomically well-developed urban or eastern areas. The mean age at diagnosis of breast cancer is 49–55 in China, which is younger than that in most western countries^19^. The age-specific incidence rate increased rapidly after the age of 25 years and peaked at the age of 45–59 years. In 2015, 31.30% of patients with breast cancer were 60 years or older in China, whereas the proportion in the USA was 56%^20^; by 2030, 41.37% of patients with breast cancer in China are estimated to be 60 years or older. The most common morphology of breast cancer was invasive ductal carcinoma, followed by invasive lobular carcinoma, results similar to those in other countries^21^. The temporal trends in age-standardized incidence rates continued to increase over the period of 2000–2015, particularly in rural areas, and will increase by approximately 11.09% between 2015 and 2030.

Several studies have shown that the increasing incidence of breast cancer in transitioning countries parallels the increases in risk factors associated with economic development and urbanization, including obesity, physical inactivity, delayed childbearing, nulliparity, prolonged endogenous hormonal exposure, and reduced duration of breastfeeding^8,22–27^. During the past several decades, China, like other developed countries, has undergone rapid economic development, cultural, and sociodemographic changes. These transformations have also led to various lifestyle changes. According to the China Health and Nutrition Survey’s findings, the traditional dietary style has been changing to a “Westernized” diet (high fat and energy density)^26^, thus leading to high rate of obesity^25^, even in adolescents^28^.

Changes in reproductive patterns that affect lifetime exposure to estrogen have a particularly crucial role in the development of breast cancer. China implemented the Chinese Family Planning Program (in which each family had only one child) in the late 1970s. The total fertility rate decreased sharply^29^ and might have affected breast cancer risk factors. Although the Chinese government initiated a two-child policy across the country in 2015 to ease the continuing decline in fertility, the incidence of breast cancer will continue to increase in the future, particularly in the recent birth cohort, because young people increasingly choose nulliparity, delay the first child birth, and reduce breastfeeding time^8,26,30^. Use of hormonal replace treatment is also an important risk factor in breast cancer. In some developed countries, the breast cancer incidence began to decrease in the early 2000s with a decline in the use of postmenopausal hormonal treatment^31^. However, owing to a lack of representative data on the use of hormonal treatment in China, the influence of this treatment on breast cancer incidence remains unclear. Against this backdrop, multi-level preventive interventions based on changeable etiologic factors might be the most effective approaches to prevent breast cancer.

Breast cancer was the fifth leading cause of cancer death in women in China, 2015. The age-standardized mortality rate was 6.48/100,000, which is lower than those in Western countries^1^. The age-specific mortality rate increased rapidly after the age of 25 years and peaked in the 80-year age group, findings similar to those in previous studies^4,32,33^. Urban areas had the highest mortality rates, but rural areas had the highest M/I, thus indicating disparities in the distribution of medical resources. The mortality rate trend increased modestly during 2000–2015. We predict that the total age-standardized mortality rate will increase by 15.60% between 2015 and 2030. Notably, in the Joinpoint analysis, we found a declining trend in mortality rate in the 40–49 age group and an increasing trend in the incidence rate in the 60–69 age group in urban areas, in contrast to the trends that we predicted with the BAMP model, probably because the Joinpoint analysis did not combine the entire age-period-cohort effect.

In Western countries, the overall mortality rate declined 39% in 1989–2015, probably because of early detection by mammography and improvements in treatment (e.g., adjuvant chemotherapy and hormonal therapy in the 1980s and targeted therapies in the 1990s). In China, the first large scale breast cancer screening program was initiated in 2005 but ended because of budget constraints^34^. Despite these obstacles, national guidelines established in 2007 recommend annual mammography for women 40–49 years of age and every 1–2 years thereafter for women aged 50–69 years of age. However, the benefits of screening remain unclear in Chinese women^35,36^, because more than 50% patients with breast cancer are under the age of 50. A lack of public health awareness is also an obstacle to screening, particularly in older women and those from areas with low socioeconomic status^37^.

Delayed early detection and treatment for newly diagnosed breast cancer also lead to poor prognosis. A nationwide multicenter study has reported that the proportions of patients diagnosed at stages I–IV are 15.7%, 44.9%, 18.7%, and 2.4%, respectively^38^. However, many women from areas with low socioeconomic status are diagnosed in stage III and stage IV^38^, and the proportion in stage IV may be substantially underestimated because most of the data are from surgical departments. Long wait times before the first treatment for newly diagnosed breast cancer, particularly if the delay leads to stage progression, or to more treatment complications, are of prognostic concern. One study^39^ has shown that, compared with waiting times of less than 2 weeks before initiation of surgical treatment, waiting times of more than 6 weeks are associated with much lower 5-year survival (90% *vs.* 80%). Given the disparities in medical resources and a lack of health awareness, large delays in diagnosis have occurred in China, particularly in less developed areas. The median time from onset of symptoms to visiting a doctor is approximately 3 months in less developed western and middle regions, as compared with 1 month in eastern areas^40^. A robust equitable health care system and multidisciplinary cooperation are urgently needed to provide ideal management of breast cancer.

The strengths of this study are the updated systematic statistics for breast cancer in China in 2015, estimated on the basis of 368 cancer registries covering a population of approximately 310 million, and these are the most representative data available to date. The temporal trends in incidence and mortality from 2000 to 2015 were based on data from 22 high-quality cancer registries and the population forecasts made by the China Population and Development Research Center, thus supporting sensible predictions. Furthermore, this study used the Bayesian age-period-cohort model as a statistical model for prediction, which considers the period and birth cohort effects as proxies for events such as risk factors, which often cannot be measured directly.

There are some limitations in this study. First, the population used to predict the total breast cancer cases and deaths in urban and rural areas was from the 2019 Revision of World Population Prospects, whereas the population used predict cases and deaths in urban and rural areas was from the 2018 Revision of World Population Prospects; we adjusted the 2018 revision population in the same proportion so that the sum of the urban and rural populations was equal to the total population. Detailed population information is provided in **Supplementary Table S5**. Second, the age-specific distribution in the total population was used to obtain the age-specific population in urban and rural areas, owing to the lack of age-specific populations in different areas, thus potentially leading to underestimation of the change due to age structure in urban areas and overestimation of the rural estimate, because of the greater proportion of the aging population in urban areas than rural areas. Third, the total change due to risk of breast cancer in rural areas may be underestimated because of their small cancer registries. However, with the acceleration of urbanization, breast cancer incidence in rural areas will increase rapidly but is not expected to exceed the incidence in urban areas.

## Conclusions

Breast cancer is the most prevalent cancer in women in China. The burden of breast cancer will continue to increase in the future. Screening programs suitable for the Chinese population urgently need to be established. The health awareness, detection, diagnosis, and treatment of breast cancer also must be improved. Financial and medical resources should be strengthened for less developed areas.
