# Supplementary material for: Breast cancer incidence and mortality in women in China: temporal trends and projections to 2030
Source: Cancer Biol Med. 2021 Aug 15;18(3):900–9. doi: 10.20892/j.issn.2095-3941.2020.0523 (PMC8330522; doi:10.20892/j.issn.2095-3941.2020.0523)
Supplement: Supplementary file 1 [file cbm-18-900-s001.pdf]

# Supplementary materials

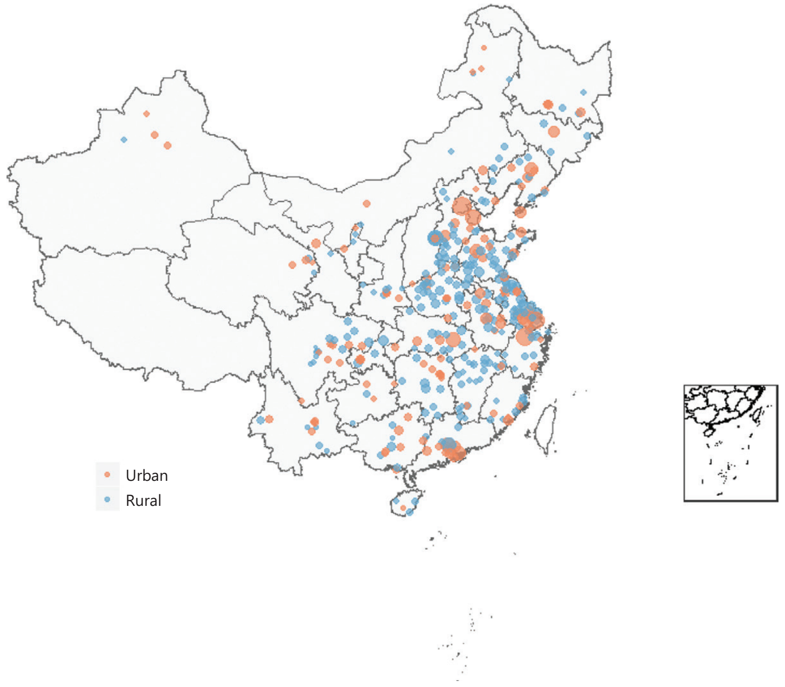


**Figure S1** Distribution of 368 cancer registries in China. The map was obtained from Basic Geographic Information Data of China.

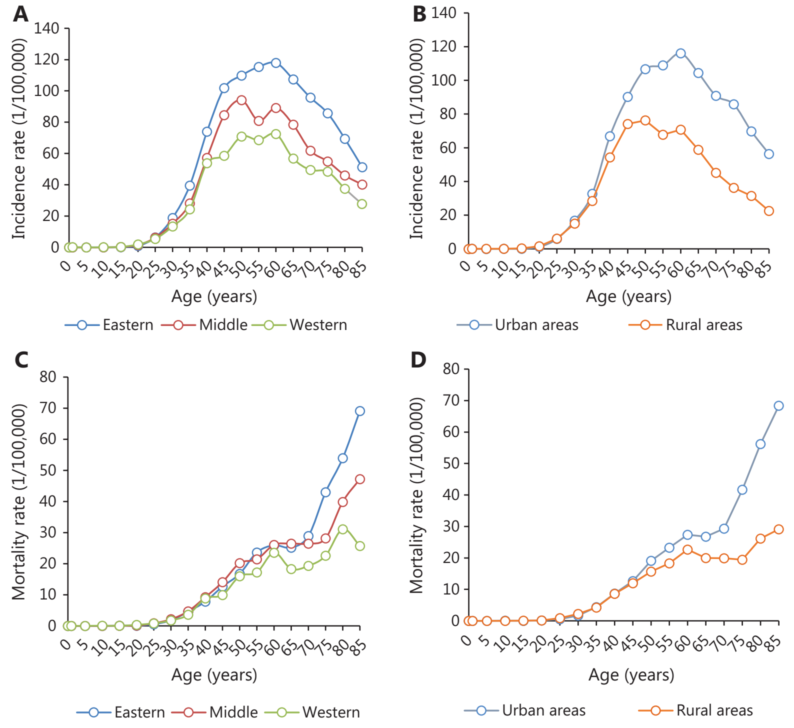


**Figure S2** Age-specific incidence and mortality of breast cancer in women in different areas in China, 2015. (A and B) Incidence in the different areas; (C and D) mortality in the different areas.

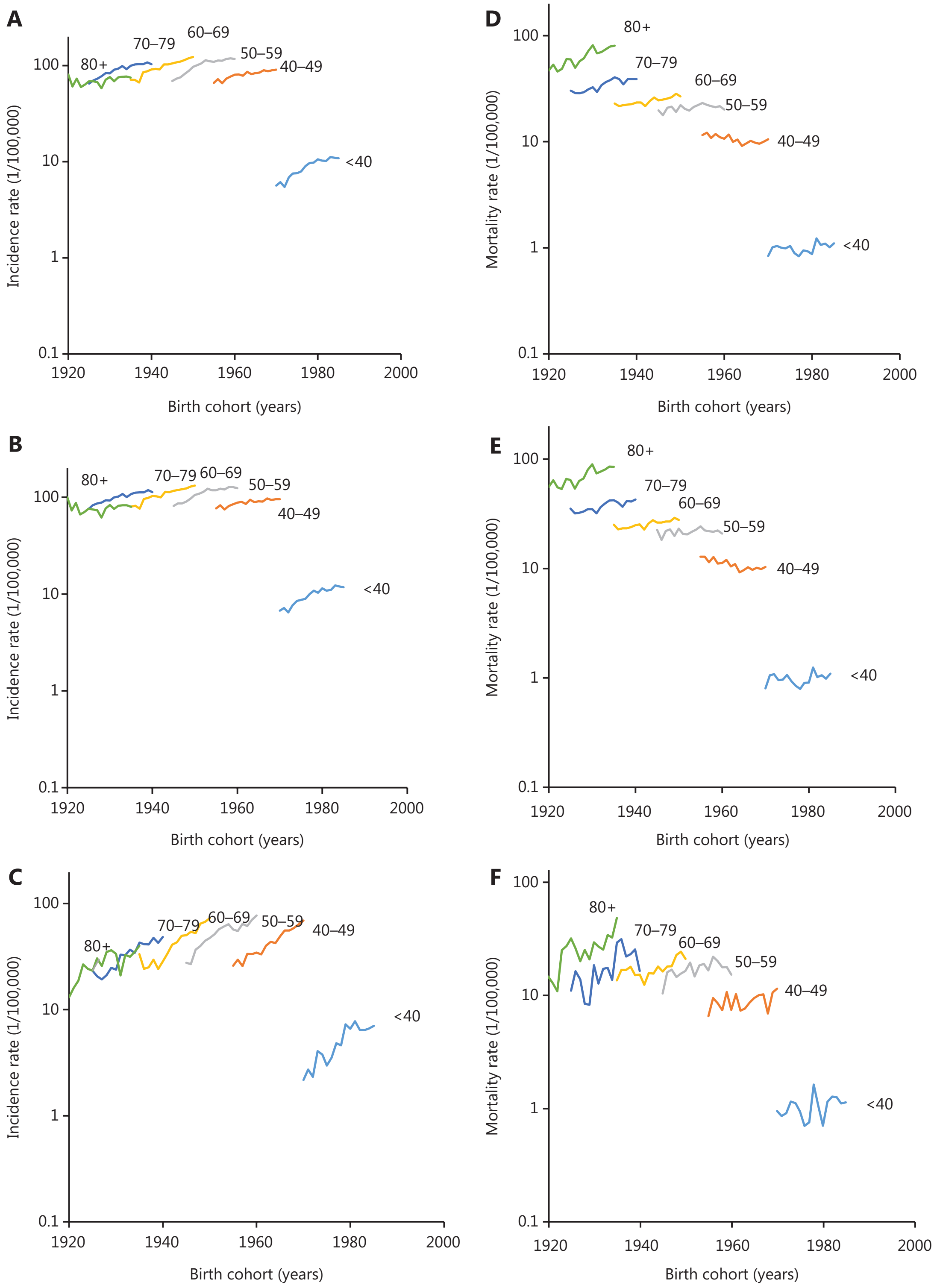


**Figure S3** Breast cancer incidence and mortality rates with different birth cohorts by age group in China. (A) Total incidence; (B) urban incidence; (C) rural incidence; (D) total mortality; (E) urban mortality; (F) rural mortality.

**Table S1** Quality control index of breast cancer in women in China in 2015

| Areas | M/I | MV% | DCO% | UB% |
|---|---|---|---|---|
| Total | 0.24 | 88.35 | 0.61 | 0.12 |
| Urban areas | 0.23 | 89.22 | 0.69 | 0.15 |
| Rural areas | 0.25 | 87.09 | 0.48 | 0.09 |
| East areas | 0.23 | 89.75 | 0.65 | 0.10 |
| Middle areas | 0.27 | 86.69 | 0.51 | 0.01 |
| West areas | 0.26 | 83.85 | 0.55 | 0.57 |

M/I, mortality to incidence rate ratio; MV%, percentage of proportion with morphological verification; DCO%, percentage of proportion with death certification only; UB%, percentage of proportion with unclear diagnosis.

**Table S2** Breast cancer incidence trends by area and age, 2000–2015

| Areas | Age (years) | Trend 1 | | Trend 2 | | Trend 3 | | AAPC 2000–2015 |
|---|---|---|---|---|---|---|---|---|
| | | Period | APC | Period | APC | Period | APC | |
| All areas | ASIR | 2000–2006 | 5.3* | 2006–2015 | 1.9* | | | 3.3* |
| All areas | <40 | 2000–2009 | 7.0* | 2009–2015 | 1.6* | | | 4.8* |
| All areas | 40–49 | 2000–2015 | 2.0* | | | | | 2.0* |
| All areas | 50–59 | 2000–2007 | 6.8* | 2007–2015 | 1.2* | | | 3.8* |
| All areas | 60–69 | 2000–2015 | 3.9* | | | | | 3.9* |
| All areas | 70–79 | 2000–2007 | 5.3* | 2007–2015 | 1.5* | | | 3.3* |
| All areas | 80+ | 2000–2015 | 0.8 | | | | | 0.8 |
| Urban areas | ASIR | 2000–2008 | 3.7* | 2008–2015 | 1.2* | | | 2.6* |
| Urban areas | <40 | 2000–2008 | 6.2* | 2008–2015 | 2.1 | | | 4.3* |
| Urban areas | 40–49 | 2000–2015 | 1.5* | | | | | 1.5* |
| Urban areas | 50–59 | 2000–2008 | 5.0* | 2008–2015 | 0.8 | | | 3.0* |
| Urban areas | 60–69 | 2000–2015 | 3.5* | | | | | 3.5* |
| Urban areas | 70–79 | 2000–2007 | 4.1* | 2007–2015 | 1.5* | | | 2.7* |
| Urban areas | 80+ | 2000–2015 | 0.1 | | | | | 0.1 |
| Rural areas | ASIR | 2000–2015 | 6.9* | | | | | 6.9* |
| Rural areas | <40 | 2000–2015 | 8.5* | | | | | 8.5* |
| Rural areas | 40–49 | 2000–2015 | 6.8* | | | | | 6.8* |
| Rural areas | 50–59 | 2000–2008 | 11.1* | 2008–2011 | −4.5 | 2011–2015 | 8.0* | 7.0* |
| Rural areas | 60–69 | 2000–2002 | −12.4 | 2002–2015 | 9.2* | | | 6.0* |
| Rural areas | 70–79 | 2000–2002 | −8.8* | 2002–2008 | 11.5* | 2008–2015 | 3.8* | 5.0* |
| Rural areas | 80+ | 2000–2015 | 5.5* | | | | | 5.5* |

APC, annual percentage change; AAPC, average annual percentage change; ASIR, age-standardized incidence rate using Segi's world standard population; *, The APC or AAPC is significantly different from zero ($P < 0.05$).

**Table S3** Breast cancer incidence trends by histological type, 2008–2015

| Histological subtype | Trend 1 | | Trend 2 | | AAPC |
|---|---|---|---|---|---|
| | Period | APC | Period | APC | 2008–2015 |
| Invasive ductal carcinoma | 2008–2012 | 5.6* | 2012–2015 | −1.7 | 3.9* |
| Invasive lobular carcinoma | 2008–2011 | 7.1 | 2011–2015 | −6.8 | −1.1 |
| Paget's disease | 2008–2015 | 12.1* | | | 12.1* |
| Medullary carcinoma | 2008–2015 | −11.2* | | | −11.2* |

APC, annual percentage change; AAPC, average annual percentage change; ASMR, age-standardized mortality rate using Segi's world standard population; *, The APC or AAPC is significantly different from zero ($P$ < 0.05).

**Table S4** Breast cancer mortality trends by area and age, 2000–2015

| Areas | Age (years) | Trend 1 | | Trend 2 | | Trend 3 | | AAPC 2000–2015 |
|---|---|---|---|---|---|---|---|---|
| | | Period | APC | Period | APC | Period | APC | |
| All areas | ASMR | 2000–2015 | 1.0* | | | | | 1.0* |
| All areas | <40 | 2000–2015 | 0.9 | | | | | 0.9 |
| All areas | 40–49 | 2000–2015 | −1.3* | | | | | −1.3* |
| All areas | 50–59 | 2000–2015 | 0.7 | | | | | 0.7 |
| All areas | 60–69 | 2000–2015 | 1.5* | | | | | 1.5* |
| All areas | 70–79 | 2000–2015 | 2.4* | | | | | 2.4* |
| All areas | 80+ | 2000–2015 | 3.8* | | | | | 3.8* |
| Urban areas | ASMR | 2000–2015 | 0.6* | | | | | 0.6* |
| Urban areas | <40 | 2000–2015 | 0.7 | | | | | 0.7 |
| Urban areas | 40–49 | 2000–2015 | −1.8* | | | | | −1.8* |
| Urban areas | 50–59 | 2000–2015 | 0.3 | | | | | 0.3 |
| Urban areas | 60–69 | 2000–2015 | 1.3* | | | | | 1.3* |
| Urban areas | 70–79 | 2000–2015 | 1.9* | | | | | 1.9* |
| Urban areas | 80+ | 2000–2015 | 3.2* | | | | | 3.2* |
| Rural areas | ASMR | 2000–2015 | 2.7* | | | | | 2.7* |
| Rural areas | <40 | 2000–2015 | 1.7 | | | | | 1.7 |
| Rural areas | 40–49 | 2000–2015 | 1.6 | | | | | 1.6 |
| Rural areas | 50–59 | 2000–2015 | 2.0* | | | | | 2.0* |
| Rural areas | 60–69 | 2000–2002 | 13.3 | 2002–2006 | −6.4 | 2006–2015 | 6.0* | 3.5 |
| Rural areas | 70–79 | 2000–2015 | 5.8* | | | | | 5.8* |
| Rural areas | 80+ | 2000–2015 | 6.3* | | | | | 6.3* |

APC, annual percentage change; AAPC, average annual percentage change; ASMR, age-standardized mortality rate using Segi's world standard population; *, The APC or AAPC is significantly different from zero ($P$ < 0.05).

**Table S5** Annual population of women in 10-year age groups, by region, 2016–2030 in China (thousands)

| Region | Year | <40 | 40–49 | 50–59 | 60–69 | 70–79 | 80+ |
|---|---|---|---|---|---|---|---|
| Total | 2016 | 338,327 | 128,008 | 97,564 | 70,226 | 35,025 | 18,848 |
| Total | 2017 | 340,077 | 128,670 | 98,069 | 70,589 | 35,207 | 18,946 |
| Total | 2018 | 341,755 | 129,305 | 98,553 | 70,938 | 35,380 | 19,039 |
| Total | 2019 | 343,324 | 129,899 | 99,005 | 71,263 | 35,543 | 19,127 |
| Total | 2020 | 344,757 | 130,441 | 99,419 | 71,561 | 35,691 | 19,207 |
| Total | 2021 | 346,045 | 130,928 | 99,790 | 71,828 | 35,824 | 19,278 |
| Total | 2022 | 347,187 | 131,360 | 100,120 | 72,065 | 35,943 | 19,342 |
| Total | 2023 | 348,193 | 131,741 | 100,410 | 72,274 | 36,047 | 19,398 |
| Total | 2024 | 349,078 | 132,076 | 100,665 | 72,458 | 36,138 | 19,447 |
| Total | 2025 | 349,858 | 132,371 | 100,890 | 72,619 | 36,219 | 19,491 |
| Total | 2026 | 350,530 | 132,625 | 101,084 | 72,759 | 36,289 | 19,528 |
| Total | 2027 | 351,093 | 132,838 | 101,246 | 72,876 | 36,347 | 19,560 |
| Total | 2028 | 351,552 | 133,012 | 101,378 | 72,971 | 36,395 | 19,585 |
| Total | 2029 | 351,914 | 133,149 | 101,483 | 73,046 | 36,432 | 19,605 |
| Total | 2030 | 352,185 | 133,251 | 101,561 | 73,103 | 36,460 | 19,621 |
| Urban | 2016 | 182,920 | 76,926 | 59,101 | 40,010 | 20,351 | 11,035 |
| Urban | 2017 | 187,834 | 78,993 | 60,689 | 41,084 | 20,897 | 11,331 |
| Urban | 2018 | 192,641 | 81,014 | 62,242 | 42,136 | 21,432 | 11,621 |
| Urban | 2019 | 197,308 | 82,977 | 63,750 | 43,157 | 21,951 | 11,903 |
| Urban | 2020 | 201,813 | 84,872 | 65,205 | 44,142 | 22,453 | 12,175 |
| Urban | 2021 | 206,142 | 86,692 | 66,604 | 45,089 | 22,934 | 12,436 |
| Urban | 2022 | 210,288 | 88,435 | 67,943 | 45,996 | 23,395 | 12,686 |
| Urban | 2023 | 214,250 | 90,102 | 69,223 | 46,862 | 23,836 | 12,925 |
| Urban | 2024 | 218,034 | 91,693 | 70,446 | 47,690 | 24,257 | 13,153 |
| Urban | 2025 | 221,645 | 93,212 | 71,613 | 48,480 | 24,659 | 13,371 |
| Urban | 2026 | 225,079 | 94,656 | 72,722 | 49,231 | 25,041 | 13,578 |
| Urban | 2027 | 228,332 | 96,024 | 73,773 | 49,942 | 25,403 | 13,774 |
| Urban | 2028 | 231,406 | 97,317 | 74,767 | 50,615 | 25,745 | 13,960 |
| Urban | 2029 | 234,304 | 98,535 | 75,703 | 51,249 | 26,067 | 14,135 |
| Urban | 2030 | 237,030 | 99,682 | 76,584 | 51,845 | 26,371 | 14,299 |
| Rural | 2016 | 155,254 | 51,154 | 38,526 | 30,219 | 14,683 | 7,819 |
| Rural | 2017 | 151,642 | 49,964 | 37,630 | 29,516 | 14,341 | 7,637 |
| Rural | 2018 | 148,071 | 48,787 | 36,744 | 28,821 | 14,003 | 7,458 |
| Rural | 2019 | 144,540 | 47,623 | 35,868 | 28,134 | 13,670 | 7,280 |
| Rural | 2020 | 141,047 | 46,473 | 35,001 | 27,454 | 13,339 | 7,104 |
| Rural | 2021 | 137,595 | 45,335 | 34,144 | 26,782 | 13,013 | 6,930 |
| Rural | 2022 | 134,192 | 44,214 | 33,300 | 26,119 | 12,691 | 6,759 |
| Rural | 2023 | 130,848 | 43,112 | 32,470 | 25,469 | 12,375 | 6,590 |
| Rural | 2024 | 127,576 | 42,034 | 31,658 | 24,832 | 12,065 | 6,425 |
| Rural | 2025 | 124,383 | 40,982 | 30,866 | 24,210 | 11,763 | 6,265 |
| Rural | 2026 | 121,274 | 39,958 | 30,094 | 23,605 | 11,469 | 6,108 |
| Rural | 2027 | 118,251 | 38,962 | 29,344 | 23,017 | 11,183 | 5,956 |
| Rural | 2028 | 115,316 | 37,995 | 28,616 | 22,445 | 10,906 | 5,808 |
| Rural | 2029 | 112,474 | 37,058 | 27,910 | 21,892 | 10,637 | 5,665 |
| Rural | 2030 | 109,728 | 36,154 | 27,229 | 21,358 | 10,377 | 5,526 |

**Table S5** Continued

**Table S6** List of 368 cancer registries

| Cancer registries | Cancer registries | Cancer registries |
|---|---|---|
| Beijing Shi | Huai'an Qu, Huai'an Shi | Tengzhou Shi |
| Rural areas of Beijing Shi | Huaiyin Qu, Huai'an Shi | Guangrao Xian |
| Tianjin Shi | Qingpu Qu, Huai'an Shi | Yantai Shi |
| Rural areas of Tianjin Shi | Lianshui Xian | Zhaoyuan Shi |
| Shijiazhuang Shi | Hongze Xian | Linqu Xian |
| Rural areas of Shijiazhuang Shi | Xuyi Xian | Gaomi Shi |
| Zanhuang Xian | Jinhu Xian | Wenshang Xian |
| Xinji Shi | Tinghu Qu, Yancheng Shi | Liangshan Xian |
| Qianxi Xian | Yandu Qu, Yancheng Shi | Ningyang Xian |
| Qian'an Shi | Binhai Xian | Feicheng Shi |
| Qinhuangdao Shi | Funing Xian | Rushan Shi |
| Daming Xian | Sheyang Xian | Laicheng Qu, Laiwu Shi |
| She Xian | Jianhu Xian | Yinan Xian |
| Ci Xian | Dongtai Shi | Yishui Xian |
| Wu'an Shi | Dafeng Qu, Yancheng Shi | Junan Xian |
| Xingtai Xian | Danyang Shi | Decheng Qu, Dezhou Shi |
| Lincheng Xian | Yangzhong Shi | Gaotang Xian |
| Neiqiu Xian | Taixing Shi | Bincheng Qu, Binzhou Shi |
| Ren Xian | Hangzhou Shi | Shan Xian |
| Baoding Shi | Jiangdong Qu, Ningbo Shi | Juye Xian |
| Wangdu Xian | Cixi Shi | Xiangfu Qu, Kaifeng Shi |
| Anguo Shi | Lucheng Qu, Wenzhou Shi | Luoyang Shi |
| Xuanhua Xian | Jiaxing Shi | Mengjin Xian |
| Zhangbei Xian | Jiashan Xian | Xin'an Xian |
| Shuangqiao Qu, Chengdu Shi | Haining Shi | Luanchuan Xian |
| Cangzhou Shi | Changxing Xian | Song Xian |
| Haixing Xian | Shangyu Qu, Shaoxing Shi | Ruyang Xian |
| Yanshan Xian | Yongkang Shi | Yiyang Xian |
| Jizhou Shi | Kaihua Xian | Luoning Xian |
| Yangquan Shi | Daishan Xian | Yanshi Shi |
| Pingshun Xian | Xianju Xian | Lushan Xian |
| Yangcheng Xian | Longquan Shi | Linzhou Shi |
| Shouyang Xian | Hefei Shi | Hebi Shi |
| Chifeng(Ulanhad) Shi | Changfeng Xian | Huixian Shi |
| Aohan Qi, Chifeng Shi | Feidong Xian | Hualong Qu, Puyang Shi |

**Table S6** Continued

| Cancer registries | Cancer registries | Cancer registries |
|---|---|---|
| Kailu Xian | Feixi Xian | Puyang Xian |
| Naiman Qi | Lujiang Xian | Yuzhou Shi |
| Hailar Qu, Hulun Buir Shi | Chaohu Shi | Yuanhui Qu, Luohe Shi |
| Arun Qi | Wuhu Shi | Yancheng Qu, Luohe Shi |
| Ewenkizu Zizhiqi | Bengbu Shi | Shaoling Qu, Luohe Shi |
| Yakeshi Shi | Ma'anshan Shi | Sanmenxia Shi |
| Genhe Shi | Tongling Shi | Fangcheng Xian |
| Linhe Qu, Bayannur Shi | Yi'an Qu, Tongling Shi | Neixiang Xian |
| Xilin Hot Shi | Tianchang Shi | Sui Xian |
| Shenyang Shi | Yingdong Qu, Fuyang Shi | Yucheng Xian |
| Faku Xian | Yongqiao Qu, Suzhou Shi | Shihe Qu, Xinyang Shi |
| Dalian Shi | Lingbi Xian | Luoshan Xian |
| Zhuanghe Shi | Shou Xian | Shenqiu Xian |
| Anshan Shi | Mengcheng Xian | Dancheng Xian |
| Benxi Shi | Jing Xian | Xiping Xian |
| Dandong Shi | Fuqing Shi | Jiyuan Shi |
| Donggang Shi | Changle Shi | Wuhan Shi |
| Yingkou Shi | Xiamen Shi | Daye Shi |
| Fuxin Shi | Tong'an Qu, Xiamen Shi | Yunyang Qu, Shiyan Shi |
| Zhangwu Xian | Hanjiang Qu, Putian Shi | Yichang Shi |
| Liaoyang Xian | Yong'an Shi | Wufeng Tujiazu Zizhixian |
| Dawa Xian | Hui'an Xian | Jingshan Xian |
| Jianping Xian | Changtai Xian | Zhongxiang Shi |
| Dehui Shi | Xinluo Qu, Longyan Shi | Yunmeng Xian |
| Jilin Shi | Yongding Qu, Longyan Shi | Gong'an Xian |
| Meihekou Shi | Wanli Qu, Nanchang Shi | Honghu Shi |
| Yanji Shi | Xinjian Qu, Nanchang Shi | Macheng Shi |
| Daoli Qu, Harbin Shi | Xunyang Qu, Jiujiang Shi | Jiayu Xian |
| Nangang Qu, Harbin Shi | Wuning Xian | Tongcheng Xian |
| Xiangfang Qu, Harbin Shi | Zhanggong Qu, Ganzhou Shi | Enshi Shi |
| Shangzhi Shi | Gan Xian | Tianmen Shi |
| Boli Xian | Dayu Xian | Furong Qu, Changsha Shi |
| Mudanjiang Shi | Shangyou Xian | Tianxin Qu, Changsha Shi |
| Hailin Shi | Chongyi Xian | Yuelu Qu, Changsha Shi |
| Shanghai Shi | Longnan Xian | Kaifu Qu, Changsha Shi |

**Table S6** Continued

| Cancer registries | Cancer registries | Cancer registries |
|---|---|---|
| Wuxi Shi | Xiajiang Xian | Shifeng Qu, Zhuzhou Shi |
| Jiangyin Shi | Anfu Xian | Hengdong Xian |
| Changzhou Shi | Wanzai Xian | Shaodong Xian |
| Liyang Shi | Shanggao Xian | Yueyanglou Qu, Yueyang Shi |
| Jintan Shi, Changzhou Shi | Jing'an Xian | Wuling Qu, Changde Shi |
| Suzhou Shi | Le'an Xian | Cili Xian |
| Changshu Shi | Yihuang Xian | Ziyang Qu, Yiyang Shi |
| Zhangjiagang Shi | Dongxiang Xian | Linwu Xian |
| Kunshan Shi | Xinzhou Qu, Shangrao Shi | Dao Xian |
| Taicang Shi | Guangfeng Qu, Shangrao Shi | Xintian Xian |
| Nantong Shi | Yanshan Xian | Mayang Miaozu Zizhixian |
| Hai'an Xian | Hengfeng Xian | Lianyuan Shi |
| Rudong Xian | Yugan Xian | Guangzhou Shi |
| Qidong Shi | Poyang Xian | Rural areas of Guangzhou Shi |
| Rugao Shi | Wannian Xian | Wengyuan Xian |
| Haimen Shi | Wuyuan Xian | Nanxiong Shi, Shaoguan Shi |
| Lianyungang Shi | Jinan Shi | Shenzhen Shi |
| Ganyu Qu, Lianyungang Shi | Zhangqiu Shi | Zhuhai Shi |
| Donghai Xian | Qingdao Shi | Nanhai Shi, Foshan Shi |
| Guanyun Xian | Huangdao Qu, Qingdao Shi | Shunde Shi, Foshan Shi |
| Guannan Xian | Linzi Qu, Zibo Shi | Jiangmen Shi |
| Shihezi Shi | Yiyuan Xian | Duanzhou Qu, Zhaoqing Shi |
| Sihui Shi | Heshan Shi | Langzhong Shi |
| Dongguan Shi | Fusui Xian | Changning Xian |
| Zhongshan Shi | Wuzhishan Shi | Guang' an Qu, Guang' an Shi |
| Luoding Shi | Qionghai Shi | Dazhu Xian |
| Jiangnan Qu, Nanning Shi | Changjiang Lizu Zizhixian | Yucheng Qu, Ya'an Shi |
| Xixiangtang Qu, Nanning Shi | Lingshui Lizu Zizhixian | Mingshan Qu, Ya'an Shi |
| Binyang Xian | Wanzhou Qu, Chongqing Shi | Yingjing Xian |
| Liuzhou Shi | Yuzhong Qu, Chongqing Shi | Hanyuan Xian |
| Guilin Shi | Shapingba Qu, Chongqing Shi | Shimian Xian |
| Wanxiu Qu, Wuzhou Shi | Jiulongpo Qu, Chongqing Shi | Tianquan Xian |
| Cangwu Xian | Jiangjin Qu, Chongqing Shi | Lushan Xian |
| Beihai Shi | Fengdu Xian | Baoxing Xian |
| Hepu Xian | Qingyang Qu, Chengdu Shi | Lezhi Xian |

**Table S6** Continued

| Cancer registries | Cancer registries | Cancer registries |
|---|---|---|
| Gangbei Qu, Guigang Shi | Longquanyi Qu, Chengdu Shi | Kaiyang Xian |
| Luocheng Mulaozu Zizhixian | Pengzhou Shi, Chengdu Shi | Huichuan Qu, Zunyi Shi |
| Hongta Qu, Yuxi Shi | Ziliujing Qu, Zigong Shi | Bijiang Qu, Tongren Shi |
| Chengjiang Xian | Renhe Qu, Panzhihua Shi | Ceheng Xian |
| Yimen Xian | Guanghan Shi | Fuquan Shi |
| Longyang Qu, Baoshan Shi | Yanting Xian | Panlong Qu, Kunming Shi |
| Tengchong Shi | Jiange Xian | Guandu Qu, Kunming Shi |
| Gejiu Shi | Chuanshan Qu, Suining Shi | Xishan Qu, Kunming Shi |
| Pingbian Miaozu Zizhixian | Shizhong Qu, Leshan Shi | Zhongwei Shi |
| Beilin Qu, Xi’an Shi | Liangzhou Qu, Wuwei Shi | Tianshan Qu, Urtimqi Shi |
| Lianhu Qu, Xi’an Shi | Xining Shi | Karamay Shi |
| Weiyang Qu, Xi’an Shi | Ledu Xian, Haidong Shi | Diqishi |
| Yanta Qu, Xi’an Shi | Huzhu Tuzu Zizhixian | Xinyuan(Künes) Xian |
| Hu Xian | Xunhua Salarzu Zizhixian | Shangzhou Qu, Shangluo Shi |
| Gaoling Qu, Xi ’an Shi | Hainan Zangzu Zizhizhou | Jingtai Xian |
| Mei Xian | Helan Xian | Huinong Qu, Shizuishan Shi |
| Long Xian | Dawukou Qu, Shizuishan Shi | Qingtongxia Shi |
| Jingyang Xian | Tongguan Xian | |
